# An Overview of Hypoglycemia in Children Including a Comprehensive Practical Diagnostic Flowchart for Clinical Use

**DOI:** 10.3389/fendo.2021.684011

**Published:** 2021-08-02

**Authors:** Alberto Casertano, Alessandro Rossi, Simona Fecarotta, Francesco Maria Rosanio, Cristina Moracas, Francesca Di Candia, Giancarlo Parenti, Adriana Franzese, Enza Mozzillo

**Affiliations:** ^1^Department of Translational Medical Science, Section of Pediatrics, Regional Center of Pediatric Diabetes, Federico II University of Naples, Naples, Italy; ^2^Department of Translational Medical Science, Section of Pediatrics, Metabolic Diseases Unit, Federico II University of Naples, Naples, Italy; ^3^Section of Metabolic Diseases, Beatrix Children’s Hospital, University Medical Centre Groningen, University of Groningen, Groningen, Netherlands; ^4^Department of Translational Medical Science, Section of Pediatrics, Federico II University of Naples, Naples, Italy; ^5^Telethon Institute of Genetics and Medicine, Pozzuoli, Italy

**Keywords:** neonatal hypoglycemia, childhood hypoglycemia, inborn errors of metabolism, endocrine hypoglycemia, glucose homeostasis, congenital hyperinsulinism

## Abstract

Hypoglycemia is the result of defects/impairment in glucose homeostasis. The main etiological causes are metabolic and/or endocrine and/or other congenital disorders. Despite hypoglycemia is one of the most common emergencies in neonatal age and childhood, no consensus on the definition and diagnostic work-up exists yet. Aims of this review are to present the current age-related definitions of hypoglycemia in neonatal-pediatric age, to offer a concise and practical overview of its main causes and management and to discuss the current diagnostic-therapeutic approaches. Since a systematic and prompt approach to diagnosis and therapy is essential to prevent hypoglycemic brain injury and long-term neurological complications in children, a comprehensive diagnostic flowchart is also proposed.

## Introduction

Hypoglycemia (HY) in pediatric age shows some peculiarities regarding its diagnosis and management, mostly linked to age dependent features in glucose homeostasis and to the broad spectrum of causes. Such causes can initially present with the same unspecific picture, but they require different treatment (1). While being frequent but hard to detect in neonatal age, it is less common in infants and toddlers, even rarer in older children (1–3). In childhood, HY is a common metabolic-endocrine emergency possibly causing permanent neurological consequences. It is therefore essential to promptly detect and treat children with HY as well as those at risk. It is crucial to appropriately investigate its specific etiology for providing adequate and specific therapy (3, 4). In this review, we present current knowledge on management of HY in neonates and children including difficulties in establishing thresholds for both definition and therapeutic intervention and providing a comprehensive overall diagnostic approach through the use of a simple practical flowchart (5–8).

## Controversies About Clinical and Biochemical Definition of Hypoglycemia

**Figure 1:**
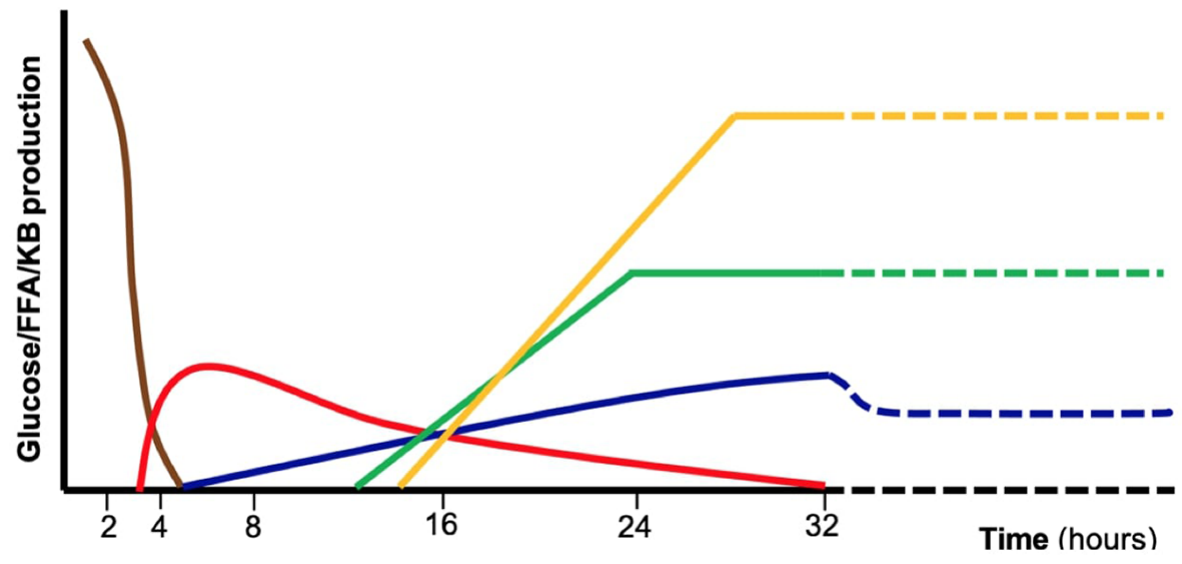
Schematic representation of the major metabolic pathways involved in glucose homeostasis during absorptive phase and fasting including exogenous carbohydrates (brown), glycogenolysis (red), gluconeogenesis (blue), fatty acid oxidation (green), ketogenesis and ketolysis (yellow). These mechanisms are tightly controlled by hormonal regulation. Defects in specific enzymes or transporters involved in those pathways as well as endocrine disorders may result in fasting intolerance and hypoglycemia. FFA, free fatty acids, KB, ketone bodies.

Glucose is the primary energy source for central nervous system metabolism, independently from the feeding state (1). Several metabolic pathways cooperate to ensure normal blood glucose concentrations in the fasted state (**Figure 1**). Such pathways are tightly regulated by the hormonal (insulin, glucagon, cortisol, and growth hormone) and autonomic (catecholamines) response. In case of impaired metabolic pathways and/or altered hormonal regulation, glucose could become too low to satisfy neuronal demand, causing classical symptoms of HY. In pediatric age, both glucose homeostasis and clinical presentation of HY show peculiarities compared to adults. In newborns, the adaptation to extrauterine life, characterized by immature hormonal and enzymatic pathways, and the higher glucose requirement of the brain, lead to a higher HY risk compared to older children and adults (9). Infants and children, have less glycogen storage and a higher substrates demand.

HY definition remains controversial in neonates and children. Some approaches define HY on the basis of symptoms, others on the plasma glucose value. In adolescents and adults, HY definition is based on the so-called “Whipple triad” [(I) symptoms of HY, (II) blood glucose level below 60 mg/dl, (III) resolutions of symptoms after glucose intake]. This definition appears inadequate for neonates and children in which symptoms are often subtle, and with the child being unable to communicate them (10). In addition, it is difficult to identify a single plasma glucose (PG) value below which symptoms of HY appear: in fact, symptoms appearance depends on additional factors, including the availability of alternative energy substrates (e.g. ketone bodies) and the severity, duration, and recurrence of low PG (7). Neurogenic symptoms are secondary to the neuroendocrine response, while neuroglycopenic symptoms are due to the low glucose availability to the brain. In neonates and infants, neurogenic and neuroglycopenic symptoms are not specific for HY. Therefore HY can be defined as the individualized condition in which PG concentration is low enough to cause symptoms and/or signs of impaired brain function (11).

Based on the above-mentioned considerations, three age-based different clinical scenarios exist:

- *Neonates <48 h of life*: signs or symptoms of neonatal HY may vary from severe (e.g. lethargy, tachypnea, hemodynamic instability, apnea, seizures, or even cardiac arrest) to milder (e.g. abnormal cry, decreased feeding, jitteriness, irritability, pallor, cyanosis, hypothermia, or diaphoresis) (12). In neonates showing specific symptoms, HY is diagnosed when PG is lower than a specific threshold: 47 mg/dl according to American Academy of Pediatrics (AAP) (13, 14), and 50 mg/dl according to Pediatric Endocrine Society (PES) (7). A different glucose threshold has been proposed for pre-term newborns (15).
- *Neonates >48 h of life, infants, and younger children unable to communicate*: HY is defined as PG <50–70 mg/dl (i.e. the normal threshold for neurogenic responses). Notably, recurrent PG levels in this range may cause the HY-associated autonomic failure, that in turn can attenuate HY autonomic symptoms (HY unawareness). Conflicting results on the definition of a safety glucose target level have emerged (16, 17). Currently, an acceptable threshold for this group is still considered 60 mg/dl (7).
- *Older children able to communicate their symptoms*: for children who are able to communicate their symptoms, Whipple’s triad could be adopted. In this age the clinical presentation is characterized by more specific symptoms as compared to neonates. In particular, the neuroglycopenic symptoms, due to the scarce availability of glucose for the central nervous system, and so indicative for lower PG level, are more clearly recognizable.

Signs and symptoms of HY are summarized and distinguished in neonatal and infant setting (**Table 1**). Neonatal HY cut off needing for intervention according with AAP, PES, BAPM are summarized in **Table 2**.

**Table 1:** Symptoms and signs of HY.

Age	Neuroglycopenic (PG < 30 mg/dl)	Neurogenic (PG < 55–65 mg/dl)
Newborn	Poor suck or poor feeding, weak or high–pitched cry, change in level of consciousness (lethargy, coma), seizures, hypotonia.	Jitteriness/tremors, pallor, sweating, irritability, tachypnea.
Infant–Child	Warmth, weakness, difficulty thinking, confusion, tiredness, drowsiness, coma, death.	*Cholinergic system*: sweating, hunger, tingling.
		*Adrenergic system*: shakiness, tremulousness, heart pounding, nervousness, anxiety.

**Table 2:** Neonatal HY Cut Off needing for intervention according with AAP, PES, BAPM.

Guidelines	Asymptomatic	Symptomatic
American Academy of Pediatrics 2011	0–4 h: <25 mg/dl on two consecutive occasion	40 mg/dl
	4–24 h: <35 mg/dl on two consecutive occasion	
Pediatric Endocrine Society 2015	0–48 h: <50 mg/dl	No clear cut off
	>48 h: >60 mg/dl	
British Association of Perinatal Medicine	Single value <18 mg/dl or two consecutive values <36 mg/dl	45 mg/dl

Modified from Kallem et al. (18).

## Management

During the first 48 h of life many healthy neonates could experience low PG, as a consequence of the physiologic adaptation to the extrauterine life. Sometimes it could last up to weeks being clinically relevant. So, it is crucial to identify as soon as possible neonates at risk of developing pathological low PG, and possibly distinguish between persistent and transient forms. Even though there is no consensus recommendation, HY persisting beyond the first 48 h, suggests a high risk of permanent pathology. Some authors consider the persistence over 7 days of HY and/or the need for more than 10 mg/kg/minute of intravenous glucose infusion as indicative for persistent HY and anamnestic and clinical features could help to identify neonates at risk of persistent HY (18). Various glucose monitoring schemes have been proposed (**Table 3**) (18). A more practical and concise indication is that infants of diabetic mothers and LGA should be screened for 12 h after birth while SGA and preterm neonates should be screened for the first 24 h (14). Besides receiving a close glucose monitoring, at-risk neonates should be fed as soon as possible preferably with breast milk because it promotes ketogenesis, an alternate source of energy for the brain (19). Despite several interventional studies support the efficacy of the preventive use of dextrose oral gel, prior to the first hour (20–23), there is conflicting evidence regarding its ability to prevent the need for intravenous glucose infusion (24–27). Indeed, prompt treatment of HY is needed to quickly restore brain demand. Based on the severity of clinical manifestations, glucose should be administered intravenously or orally. Similarly to the diagnosis, several interventional thresholds for HY have been proposed (18, 28).

**Table 3:** Glucose monitoring for neonates at risk for HY.

Type of HY	Onset	Duration	HY degree	Response to glucose	Duration of monitoring	Examples
Early transitional adaptive	<6–12 h	12–24h	Mild	Good	24–48 h	Preterm infants, infant of diabetic mother, intrapartum glucose infusion, hypothermia
Secondary	12–24 h	24–48 h	Mild	Good	24–48 h	Asphyxia, sepsis, intraventricular bleeding
Classic transient neonatal	24–48 h	48–72 h or more	Moderate to severe.	Requires often higher glucose infusion rate	48–72 h	Small for gestation
			About 80% are symptomatic			
Severe recurrent	Variable	>7 d	Severe	Requires higher glucose infusion rates >10–12	May be days to weeks	Congenital hyperinsulinism, metabolic and endocrine forms

Modified from Kallem et al. (18).

Two major age-based groups can be distinguished:

- *Neonates*: asymptomatic neonates who cannot maintain PG above 50 mg/dl (threshold for neuroglycopenic symptoms) after the first 48 h could be at risk of a persistent HY disorder. A structured intervention can only be indicated if the patient is symptomatic or has PG <60 mg/dl (threshold for neurogenic symptoms).
- *Infants and children*: in case of mild to moderate HY, in infants/children able to take simple sugars orally, oral glucose has the quickest response (even in case of unknown etiology). Compared to sucrose, glucose leads to higher and earlier glycemic peak (29). Notably, in some metabolic conditions [e.g. hereditary fructose intolerance or defects in neoglucogenesis as Fructose 1,6 bisphosphatase (FBPase) deficiency] the administration of sugary drinks containing sucrose could considerably worsen the metabolic decompensation. In such conditions or in case of unknown etiology 10–20 grams of oral glucose are recommended, followed by a snack of starchy carbohydrates or a milk feed in infants (1, 30). In severe cases (when the patient is unconscious/unable to take anything orally) in an out of hospital setting and/or in the case of unavailable venous access, unless a diagnosis of a specific Inborn Metabolic Disorder (IMD) [e.g. Glycogen storage disease (GSD) type I] has been performed, glucagon (1 mg for children aged more than 12 years and/or weighing at least 25 kg, while 0.5 mg for younger/leaner) (1) should be used, due to its fast counter-insular action. Glucagon should be administered carefully because repeated/excessive doses may induce vomiting and so aggravate HY. Furthermore, it could be ineffective in case of long lasting HY or fasting, when liver glycogen stores may have already been depleted. In this case glucose must be infused starting with a bolus of 200–500 mg/kg (2–5 ml/kg of 10% glucose solution) followed by an infusion with 10% glucose adjusted to maintain euglycemia based on the age requirements (1).

## Etiological Diagnosis

### Endocrine Causes

#### Congenital Hyperinsulinism

Congenital Hyperinsulinism (CH) represents the most common cause of persistent HY in infants and children, with an estimated incidence of 1:40.000–50.000 in general population. It is a heterogeneous and complex biochemical disorder characterized by the dysregulated insulin secretion from pancreatic β-cell causing random HY associated with low/normal ketones and absence of metabolic acidosis. Besides the classical neonatal onset, there are also late-onset forms that can appear in adolescence/adulthood (0.5–5.0% of cases) and could exhibit glycemic fluctuations from HY to hyperglycemia (31). In CH one or more steps of insulin secretion are disrupted due to a genetic defect, resulting into an insulin release that is independent from PG levels; sometimes, it is triggered by peculiar events, such as meal and exercise (31). CH genetic diagnosis could be achieved in about half of the patients. Besides syndromic conditions, currently about 14 genes are known to cause monogenic forms. Considering the known pathogenic mechanisms, currently CH could be grouped into four categories (32):

- *Channel Defects (ChD; genes ABCC8, KCNJ11, KCNQ1, CACNA1D)*: among these mutations, ABCC8 and KCNJ11 (KATP channel subunits Kir6.2 and SUR1, respectively) causes the most common and severe forms of CH especially in case of biallelic mutation, although there have been reported patients carrying *ABCC8* biallelic mutations with optimal response to Diazoxide (DZX) (33, 34) even showing progressive resolution of hypoglycemia (35).
- *Metabolic Defects (MeD; genes GLUD1, GCK, HADH, UCP2, HK1, PMM2, PGM1)*: this class includes enzyme defects causing abnormal intracellular levels of specific metabolites regulating insulin release. Among these mutations, GLUD1 activating result into increased glutamate dehydrogenase enzyme (GDH) activity and cause the Hyperinsulinism/Hyperammonemia syndrome (the second most common cause of CH). Since GDH is allosterically enhanced by Leucine, protein load can induce HY. HY due to dominantly inherited by GCK activating mutations is clinically heterogeneous with respect to severity and age of onset.
- *Transcription factors Defects (TfD; genes HNF1 α, HNF4 α, FOXA2)*: this class includes molecular defects in the transcriptional factors that regulate the glucose-induced secretion of insulin. Patients affected by these mutations are subjected to transient hyperinsulinemic HY followed by the development of Maturity Onset Diabetes of the Young during adolescence (36, 37).
- *Syndromic conditions*: CH could be a manifestation of several syndromic conditions (**Table 4**).

**Table 4:** Syndromic causes of CH.

Overgrowth syndromes	*Gene*	*Chromosome*	*Inheritance*

Beckwith–Wiedemann syndrome	IGF2/H19	11p15.5–15.4	Autosomal dominant
	CDKN1C		Sporadic
	KCNQ1OT1		Paternal uniparental disomy
Sotos syndrome	NSD1	5q35.2–35.3	Autosomal dominant
	NFIX	19p13.3	Sporadic
Simpson–Golabi–Behmel syndrome	GPC3	Xq26	X–linked
Perlman syndrome	DIS3L2	2q37	Autosomal recessive
**Chromosomal abnormality syndromes**			
Turner syndrome	KDM6A	Xp11.2	Sporadic
Trisomy 13	CDX2, IPF	Trisomy 13	Sporadic
**Postnatal growth failure syndromes**			
Kabuki syndrome	KMT2D	12q13.12	Autosomal recessive
	KDM6A	Xp11.3	Sporadic
Costello syndrome	HRAS	11p15.5	Autosomal dominant Sporadic
**Contiguous gene deletion affecting the ABCC8 gene**			
Usher syndrome	USH1C	11p15.1	Autosomal recessive
Timothy syndrome	CACNA1C	3p21.1	Autosomal dominant Sporadic
Insulin receptor mutation			
Insulin resistance syndrome (Donohue syndrome)	INSR	19p13.2	Autosomal recessive
**Congenital Disorders of Glycosylation (CDG)**			
CDG Type Ia	PMM2	16p13.2–13.3	Autosomal recessive
CDG Type Ib	PMI	15q22–24	Autosomal recessive
CDG Type Ic	hALG3	3q27	Autosomal recessive
**Other causes**			
Poland syndrome	UCMA	10p13–14	Sporadic
CHARGE syndrome	CHD7	8q12	Autosomal dominant

Modified from Galcheva S, et al. (32).

From the histopathological point of view, CH is classified into three variants (31, 38). In diffuse forms, all β-cells share the same molecular defect and show the same morphology. In focal forms, a β-cell cluster develops as a nodular adenomatous hyperplasia because of a confined molecular defect in the 11p15.1-11p15.5 imprinted region, that involve the ABCC8/KCNJ11 genes. These forms usually develop sporadically in a patient carrying a recessive ABCC8/KCNJ11 paternally inherited mutation, when a somatic loss of the maternal allele occurs (“double hit”). ABCC8 mutations can cause phenotypes who switch from HY in infancy to hyperglycemia in adolescence and even adulthood (39–41). Late onset forms are mostly linked to dominant mutations of ABCC8/KCNJ11 genes or to activating mutation of GCK gene (40). CH diagnosis may be suspected at any insulin concentration detectable in a hypoglycemic plasma sample thus as a marker of inappropriate insulin secretion (1, 42). Indirect signs of this phenomenon are the absence of ketonemia (excepted for HADH deficit) and fatty acidemia. Adjunctive diagnostic criteria for CH could be a positive response to glucagon or octreotide injection (glucose levels >1.5 mmol/L) and the need for more than 8 mg/kg/min of glucose infusion to maintain euglycemia (1, 32, 38, 40, 41). Some authors (42) have proposed a classification of CH diagnostic criteria as reported in the **Table 5**. Once the diagnosis of CH has been established, genetic test should be performed; as a general rule, ABCC8/KCNJ11 mutations must be investigated first. Concurrently to genetic tests, a prompt treatment should be started with diazoxide (DZX) and then, in case of unresponsiveness, with octreotide (38, 41, 42, 44). In fact, many ABCC8/KCNJ11 mutations cause refractoriness to DZX. These cases require an 18-Fluoro-DOPA-Positron Emission Tomography (PET) to search for any focal forms, and need to be treated with octreotide. Dosage adjustment could be required due to possible tachyphylaxis. Side effects include abdominal discomfort, diarrhea (rarely necrotizing enterocolitis), and in long term, bile sludge/gallstones and suppression of pituitary hormones. Long-acting release (LAR) octreotide analogues, administered monthly, have also been successfully tried in children (45, 46) and even preferable for better compliance and safety (47), however they take a long time to achieve the steady state (lanreotide needs 23–30 days and sandostatin 3 months to gain therapeutic blood concentration). They should initially be administered together with octreotide: the starting dose is 30–60 mg for lanreotide (subcutaneously) while for sandostatin-LAR (intramuscularly) the dose is equivalent to the cumulative 31-day subcutaneous octreotide dose, calculated by multiplying the daily dose of octreotide (5–25 µg/kg) for 31. Other drugs proposed for the treatment of DZX un-responsive cases include Nifedipine, Sirolimus, and Glucagon-like peptide-1 (GLP-1) receptor antagonist “Exendin” (1, 42). In particular, Sirolimus, is an antiproliferative drug that reduces insulin secretion probably by lowering the mammalian target of rapamycin (mTOR), a serine/threonine kinase that is overexpressed in the diffuse variant of CHI and which enhances insulin secretion; moreover, Sirolimus causes depletion of intracellular Calcium decreasing insulin release (48). This drug has shown variable efficacy and safety being even able to avoid surgery up to 18 months, but sometimes uneffective both for duration and power to reduce hypoglycemic events (49). Although there haven’t been reported major side effects, it should be used with caution for its immunosuppressive action and long-term follow-up studies are needed for chronic toxicity (42). Therapy effectiveness in CH patients is based on blood glucose monitoring. The tools used for this monitoring are the same used for the glycemic monitoring of type 1 diabetes patients (50) such as blood glucose sampling and continuous subcutaneous glucose monitoring (51). According with the most recent evidences, 18-Fluoro-DOPA PET should be offered not only in case of refractoriness to DZX, but also when no mutation is identified or in case of a single recessive paternal inherited mutation in ABCC8/KCNJ11, that could reveal focal forms, even if responsive to DZX. While surgery could be definitively curative for focal forms, near-total pancreatectomy, reserved for diffuse forms unresponsive to available drugs, could cause iatrogenic diabetes and allocate patients to life-long pancreatic enzyme replacement and insulin therapy. Moreover, the residual pancreatic tissue left near the common bile duct and along the duodenum could be responsible for persistent HY. Data from long-term follow-up show efficacy in prevention of severe hypoglycemic episodes but with only few cases of remission (52).

**Table 5:** Diagnostic criteria of CH.

**Cardinal diagnostic criteria**	Low plasma glucose (<3 mmol/L) with
	Detectable serum insulin
	Detectable C–peptide
**Biochemical criteria**	Suppressed/low beta–hydroxybutyrate and acetoacetate
	Suppressed/low serum free fatty acid
**Clinical criteria**	Increased requirement of glucose infusion rate (>8 mg/kg/min)
	Positive response to i.m./i.v. glucagon (glycemic response >1.5 mmol/L)
**Supportive criteria**	Positive response to s.c./i.v. octreotide
	Low serum levels of IGFBP–1 (suppressed by insulin)
	Suppressed branch chain amino acids
	Normal lactic acid
	Normal plasma hydroxybutyrylcarnitine
	Normal ammonia
	Appropriate counterregulatory hormone response (cortisol >20 mcg/dl, GH > 7 ng/ml)
	Provocation test (leucine loading or exercise testing) may be needed in some patients

Modified from Vora et al. (43).

### Adrenal Insufficiency

Adrenal insufficiency (AI) is a life-threatening condition in which the adrenal cortex is unable to adequately produce steroid hormones. AI can be distinguished in Primary (PAI) or Central (CAI), depending on the impairment of adrenal cortex or hypothalamus/pituitary gland respectively. The most common etiology of PAI in children is the Congenital Adrenal Hyperplasia (CAH) due to 21-hydroxylase deficiency. In PAI both glucocorticoids and mineralocorticoids synthesis are affected, while in CAI only glucocorticoid synthesis is compromised (53, 54).

Glucocorticoids, mostly cortisol, play an essential role in glucose metabolism (55): in the liver they promote glucose output by activating gluconeogenesis and triglycerides accumulation (56–59); in the muscle they suppress glucose uptake and glycogen synthesis, accelerate protein breakdown, and inhibit protein synthesis; in adipose tissue they promote lipolysis and so increase serum FFA and glycerol (60) thus, together with amino-acids coming from protein catabolism, they provide substrates for gluconeogenesis. Cortisol deficiency results in incapacity to raise up glucose levels in stressful conditions, causing HY associated with low/normal ketones and absence of metabolic acidosis.

Clinical presentation of PAI may be non-specific with anorexia, weight loss, fatigue, abdominal pains, headache, nausea, arthralgia, myalgia, joint pain in chronic forms (61) or with an “adrenal crisis,” characterized by a cardiovascular decompensation due to massive impaired electrolyte and fluid balance, in case of acute onset (61). The critical sample collected during an adrenal crisis will show hyponatremia, metabolic acidosis (normal anion gap, increased serum chloride), HY, hyperkalemia, and low cortisol level.

#### Congenital Hypopituitarism and Growth Hormone Deficiency

Congenital Hypopituitarism is a pathologic condition characterized by a partial or a total deficiency in one or more pituitary hormones (62). Among those conditions, ACTH and GH deficiency (GHD) may present with HY associated with low/normal ketones and absence of metabolic acidosis. ACTH deficiency has been already described above.

Although the cause of HY in hypopituitarism is still debated, it is known that GH and cortisol, in physiologic doses, act synergistically to elevate the blood glucose, and that the replacement of both hormones is necessary to normalize insulin secretion and maintain normal glucose homeostasis in children with hypopituitarism; probably GHD is responsible of HY because of loss of amino acid mobilization to support gluconeogenesis (63, 64).

Congenital Hypopituitarism should be suspected in neonates carrying dysmorphic features with midline defects, ocular and craniofacial anomalies, and in males, micropenis often with undescended testes. Detection of persistent HY and jaundice reinforces the suspect (65–68). GHD could also appear in evolutive age mostly with its auxological consequences: short stature, delayed bone age, decrease in the growth rate (69).

### Metabolic Disorders

#### Glycogen Storage Diseases (GSD)

All Metabolic disorders are resumed in **Table 6** and **Supplementary Table 1**. GSD are secondary to defects of the enzymes and transporters involved in glycogen breakdown and synthesis. Their overall incidence is 1:25,000 births. More than 12 GSD types are recognized. Based on clinical presentation, they are classified as hepatic GSD (e.g. GSDI) and muscle GSD (e.g. GSDII, GSDV). HY and hepatomegaly are the primary manifestation of the hepatic GSD (GSD0a, GSDI, GSDIII, GSDVI, GSDIX, GSDXI). GSDIII is the only GSD presenting with concomitant liver and muscle involvement. Based on the ketone levels hepatic GSD are traditionally defined as ketotic (GSD0a, GSDIII, GSDVI, GSDIX, GSDXI) or non-ketotic (GSD I). Genetic studies are the preferred method for diagnosing hepatic GSD (enzyme tests are performed in selected cases). Dietary plan with frequent feedings and uncooked cornstarch (UCCS) and/or tube feeding are the cornerstone of the treatment for hepatic GSD; carbohydrates are given to maintain euglycemia, but excessive carbohydrate intake may result in hyperinsulinemia with consequent complications (70). Carbohydrates restriction with protein supplementation or ketogenic diets are recommended to avoid glycogen storage and to minimize insulin secretion in some forms (71). Restricted fructose and galactose intake aims at avoiding acidosis in GSDI. Major hepatic GSD are discussed.

GSD I is the most common and severe GSD (both glycogenolysis and gluconeogenesis are impaired). It is due to a defect of either the catalytic (GSDIa, 80% of cases) or the microsomal glucose 6-phosphate transporter (GSDIb, 20% of cases) of the G6Pase system. GSDI patients usually present at 3–6 months of age with fasting HY, lactic acidosis and hypoketosis (usually 2–4 h after meal), hepatomegaly, doll-like face, failure to thrive, hyperlipidemia, and hyperuricemia. Additionally, GSDIb patients show neutropenia and recurrent infections (72). Long-term complications include liver neoplasms, renal disease, and increased risk of inflammatory bowel disease (73) and autoimmune (74, 75) or endocrine disorders (76–78).

GSDIII is due to glycogen debrancher enzyme deficiency. Two main subtypes are recognized: GSDIIIa (85% of the cases, mixed liver and muscle involvement) and GSDIIIb (15% of the cases, isolated liver involvement). As gluconeogenesis is intact, HY is usually less severe than GSDI showing prominent fasting ketosis without lactic acidosis. Transaminases concentrations are usually higher (may exceed 1,000 U/L) with less severe hyperlipidemia compared to GSDI. Bone disease (78) and benefit of a high-fat diet on muscle symptoms have been reported (79).

GSDVI and GSDIX are secondary to liver glycogen phosphorylase and glycogen phosphorylase kinase defect, respectively. They are generally mild disorders improving with age. However, they can also present with symptomatic fasting ketotic HY, hyperlipidemia, increased transaminases, hepatomegaly, growth retardation, and hypotonia (80).

GSD0 is caused by a deficiency of hepatic glycogen synthase resulting in inadequate production of hepatic glycogen. The clinical manifestations include fasting ketotic hypoglycemia accompanied by low levels of alanine and lactate and postprandial hyperglycemia and hyperlactatemia. Unlike other GSDs, patients with GSD0 usually do not develop hepatomegaly (81, 82).

GSDXI (Fanconi-Bickel syndrome) is caused by deficiency in a solute carrier family 2 protein (GLUT-2) that is expressed in hepatocytes and proximal renal tubule. Patients typically present at 3–10 months of age with hepatomegaly, Fanconi syndrome (e.g. severe glycosuria, polyuria, hyperaminoaciduria, hypophosphatemic rickets, acidosis, hypokalemia, hypochloremia), failure to thrive, fasting HY, and postprandial hyperglycemia. Only symptomatic treatment is available (frequent feeds with complex carbohydrates, electrolytes replacement, vitamin D) (5).

#### Hereditary Fructose Intolerance

HFI is caused by deficiency of Aldolase B, resulting into inhibition of gluconeogenesis (inhibition of Aldolase A) and glycogenolysis (inhibition of glycogen phosphorylase A) secondary to fructose 1-phosphate accumulation. Symptoms usually present at weaning, after the ingestion of food containing fructose, sucrose, or sorbitol (e.g. fruit, vegetables) and include post-prandial HY, with ketosis and lactic metabolic acidosis, hepatomegaly, vomiting, pallor, sweating, lethargy, failure to thrive, convulsions, and eventually coma. Acute liver failure and renal dysfunction (proteinuria, glycosuria, hyperaminoaciduria) are also observed. Most patients develop a natural aversion to fruit/sweets. Therapy includes avoidance of dietary fructose, sucrose, and sorbitol (5), although mild signs of liver injury, without progression on a long-term follow-up could be detected in patients on a FSS-free diet, particularly with specific genotypes (83).

#### Galactosemia

Classical galactosemia is caused by deficiency of galactose-1-phosphate uridyltransferase (GALT) (the enzyme converting lactose into glucose and galactose) resulting into accumulation of galactose 1-phosphate, galactitol, and galactonate in blood and tissues. Symptoms usually appear a few days after the ingestion of breast or formula milk and include vomiting, diarrhea, poor feeding, nuclear cataract, jaundice, hepatomegaly, and high transaminases evolving to liver failure (HY, bleeding tendency) and renal failure; *Escherichia coli* sepsis is common.

#### Inherited Disorders of Gluconeogenesis

The conversion of pyruvate into glucose is the central pathway of gluconeogenesis. Overall, disorders of gluconeogenesis present with recurrent HY and lactic acidosis with or without ketosis. Major inherited disorders of gluconeogenesis are described.

FBPase deficiency is a disorder of gluconeogenesis characterized by episodic acute crisis of HY, lactic acidosis (lactate may rise up to 25 mmol/L), and (usually) ketosis manifesting with hyperventilation, apneic spells, hepatomegaly (with normal transaminases), seizures, coma, and brain damage. The crises are likely to occur when glycogen reserves are limited (as in newborns or after ingestion of large amount of fructose) or exhausted (e.g. fasting, intercurrent illness) and are reversed by high glucose infusion rates (about 1.5 times maintenance). The frequency of the attacks decreases with age and patients are usually well between attacks. Treatment includes frequent feedings and avoidance of prolonged fasting (84).

Pyruvate carboxylase (PC) deficiency is a defect of both gluconeogenesis and Krebs cycle. Although fasting HY can occur, this disorder usually presents with severe encephalopathy, developmental delay, seizures, movement disorders, failure to thrive, and metabolic acidosis. A high lactate to pyruvate ratio with a low hydroxybutyrate to acetoacetate ratio is suggestive of the diagnosis. Treatments include intravenous glucose infusion, bicarbonate, dietary management, and supplementation with citrate, aspartate, dichloroacetate, biotin, and thiamine (85).

Phosphoenolpyruvate carboxykinase (PEPCK) deficiency affects gluconeogenesis and can cause HY, failure to thrive, lactic acidosis, and lipid accumulation in the kidney and liver. Only six patients have been reported in the literature and its clinical relevance is currently disputed (86).

Glycerol kinase deficiency (GKD) can present either isolated or together with congenital adrenal hypoplasia or Duchenne muscular dystrophy (partial deletion of Xp21). Patients with isolated GKD can develop episodic vomiting with HY, hyperketonemia, metabolic acidosis, and coma. Typically, high glycerol excretion in the urine is found by gas chromatography-mass spectrometry. Metabolic crises should be avoided by providing an adequate supply of fluid, calories, and glucose during intercurrent illness (87).

#### Congenital Disorders of Glycosylation

CDG constitute a group of conditions due to defects in the glycoprotein synthesis. Around 90 CDG types are currently recognized. Phosphomannomutase 2 Deficiency and Glucosyltransferase 1 Deficiency are the most common CDG. A broad spectrum of symptoms including psychomotor retardation, failure to thrive, hypotonia, deafness, bleeding tendency, cerebral hemorrhage, cardiomyopathy, hypogonadism, and HY (hyper- or normoinsulinemic) is known (88).

#### Fatty Acids Oxidation Disorders

FAODs constitute a group of conditions characterized by hypoketotic HY and presenting with great variability. Three typical presentations are known for FAOD:

- Acute hypoketotic HY with lactic acidosis and encephalopathy with hepatomegaly and liver dysfunction (including hyperammonemia); symptoms usually present under catabolic circumstances (e.g. newborn, prolonged fasting, intercurrent illness)
- (Hypertrophic) cardiomyopathy and arrhythmias
- Myopathy presenting with weakness and/or acute rhabdomyolysis with symptoms precipitated by exercise or intercurrent illness (89).

Diagnosis can be suggested by acylcarnitine profile and confirmed by enzyme testing or gene sampling (90). Treatment includes a high carbohydrate diet to maintain euglycemia and to avoid prolonged fasting or stress induced states. Carnitine supplementation can be used for specific FAOD (91).

#### Disorders of Ketone Body Metabolism

Disorders of KB metabolism can present either in the first days of life or later in childhood. Similarly to FAOD, prolonged fasting and intercurrent illness are triggers to metabolic decompensation. Ketogenesis defects are characterized by hypoketotic HY with or without hyperammonemia, metabolic acidosis, and liver disease. Decompensations lead to encephalopathy, vomiting, and coma. Conversely, ketolysis defects present with episodes of hyperketotic HY and severe ketoacidosis in childhood; patients are healthy between episodes (92).

#### Disorders of Oxidative Phosphorylation

Disorders of Oxidative Phosphorylation (OXPHOS) are clinically, biochemically, and genetically heterogeneous. They are due to mutations in nuclear genes coding for respiratory complexes subunits and can present at any age with a wide range of possible symptoms, including fasting HY with lactic acidosis and variable ketone bodies levels. Children often suffer from encephalomyopathic disease (93).

#### Organic Acidemias

OA are disorders of intermediary metabolism due to defect of enzymes involved in branched-chain amino acid catabolism. They are characterized by the mitochondrial accumulation of CoA metabolites causing metabolic acidosis, elevated lactate, ketotic HY, and hyperammonemia. The most common OA are Methylmalonic acidemia (MMA), Propionic acidemia (PA), and Isovaleric acidemia (IVA).

Three clinical presentations are recognized:

- Neonatal (intoxication type): lethargy, poor feeding, encephalopathy, myoclonic jerks, multiorgan failure.
- Chronic intermittent: episodes of ketoacidosis, lethargy, cerebral involvement.
- Chronic progressive: vomiting, failure to thrive, psychomotor retardation, hypotonia, renal disease.

OA are diagnosed by their specific urinary organic acid profiles or abnormal plasma acylcarnitines.

The diagnosis is confirmed with enzymatic studies and/or molecular DNA testing (94). OA are included in NBS programs in several countries with an increasing number of patients diagnosed pre-symptomatically (95). Treatment of the acute phase is aimed at correcting hyperammonemia (by temporary stopping protein intake, promoting anabolism, and administering ammonia scavengers), metabolic acidosis, and HY. The cornerstone of chronic treatment are protein-restricted diet, long-term ammonia scavengers, vitamin cofactors, and carnitine supplementation (carnitine transforms toxic CoA esters into less toxic carnitine esters).

#### Idiopathic Ketotic HY

Ketotic HY is the most common cause of childhood HY. It usually presents between 18 months and 5 years and resolves spontaneously by the age of 9 years. Typically, the child presents with symptomatic HY in the morning after long fast often precipitated by an intercurrent illness. Glucose <55 mg/dl and massive ketosis are observed. Metabolic acidosis can also develop. The child improves dramatically on dextrose infusion (conversely glucagon injection elicits little or no increase in glucose concentrations) and is usually restored to normal health within hours. Despite being the most common cause of HY in childhood, there are no specific diagnostic tests for ketotic HY. Therefore, all possible causes of HY must be ruled out (diagnosis of exclusion). Treatment measures include avoidance of prolonged fasting, UCCS, and close monitoring of oral intake when in stressed states (such as illness) to avoid HY (2).

## Diagnostic Pathway: Practical Approach to HY in Childhood

The diagnostic path of HY results from the combination of medical history and clinical, dietary, and biochemical data. A systematic approach is necessary to collect relevant information.

### Personal History

The first step consists in collecting information on the timing of the hypoglycemic event, including:

Age of onset (neonatal, infant, child)

- Fasting tolerance (e.g. feeding frequency, night snack, morning ketosis)
- Temporal relation with meals (fasting, post-prandial, random)
- Relation to/Avoidance of food [e.g. protein, fruit, fruit (juice), (ga)lactose]
- Associated conditions/triggers
- Recurrence (e.g. intercurrent disease, fatigue)
The following information should also be carefully detailed:
- Perinatal history: birth weight, gestational age, gestational diabetes, and any other form of perinatal
distress and perinatal glucose requirements (e.g. >10 mg/kg/min glucose intravenously).
- Growth and developmental milestones (e.g. intellectual disability, movement disorders, epilepsy)
- Family history: relatives with HY/hyperglycemia or IMD, previous miscarriages or deaths, consanguinity, medications, and social history.

### Physical Examination

Physical examination can reveal signs pointing to:

- Endocrine dysfunction, such as micropenis, short stature, midline anomalies (hypopituitarism), skin hyperpigmentation, abdominal pain, muscle pain, weight loss, signs of hyperandrogenism [Adrenal Insufficiency (AI)]
- Inherited metabolic disease, such as hepato(spleno)megaly (since the liver and spleen size become larger with age, patients’ age and height should be considered for adequate assessment), jaundice, spider angiomas (e.g. GSD), cataract (e.g. galactosemia), absence of dental caries [e.g. Hereditary Fructose Intolerance (HFI)], arrhythmias and/or heart murmur [e.g. Fatty Acid Oxidation Disorders (FAOD)], movement disorders [e.g. Organic Acidemias (OA)], hypotonia and inverted nipples and/or bleeding tendency [e.g. Congenital Disorders of Glycosylation (CDG)], multisystem involvement (e.g. mitochondrial disorders)
- Dysmorphic features, macrosomia, hemihypertrophy (e.g. CH, genetic syndromes of overgrowth)

### Laboratory Investigations

Biochemical tests can provide crucial information to reach a final diagnosis. Indeed, specific biochemical patterns can point to specific defects. In particular, low ketones at the time of hypoglycemia may immediately suggest a diagnosis of hyperinsulinism or FAOD. Contextual levels of Free Fatty Acids (FFA) could help to distinguish between endocrine or metabolic etiology, as low ketones together with low FFA suggest hyperinsulinism, while low ketones together with increased FFA suggest FAOD or defects of ketolysis (when FFA/KB ratio results below 0.3) (**Table 6**).

**Table 6:** Main clinical and biochemical features of major metabolic causes of childhood HY.

Disorder	Timing	Lactate	Ketones	Additional biochemical abnormalities	Clinical features
***Ketotic hypoglycemia***	Fasting >6 h	+/−	+	Low alanine	Fever
		M.A.			Vomiting
					Diarrhea
***Glycogen storage disease type I***	Fasting (2–4 h)	+	(−)	Elevated lipids	Hepatomegaly
		M.A.		Elevated uric acid	Doll–like face
				Elevated transaminases	
				Neutropenia (GSDIb)	
***Glycogen storage disease type III/VI/IX***	Fasting (2–6 h)	−	+	Elevated lipids	Hepatomegaly
				Elevated transaminases	Cardiomyopathy
				Elevated CK (GSDIIIa)	(GSDIIIa)
***Hereditary Fructose Intolerance***	1–2 h after the ingestion of fructose, sucrose, sorbitol	+	+/−	Elevated transaminases	Vomiting
		M.A.			Diarrhea
					Hepatomegaly
					Liver failure
					Fatty liver
***Galactosemia***	1–2 h after the ingestion of galactose, lactose	−	+/−	Elevated bilirubin	Vomiting
		M.A.		Elevated transaminases	Hepatomegaly
				Abnormal clotting tests	Liver failure
					Cataract
					Sepsis
***Fructose 1,6 bisphosphatase deficiency***	Fasting >6 h	+	+	Elevated alanine	Intercurrent disease
		M.A.		Elevated pyruvate and glycerol 3–phosphate	Hepatomegaly
***Pyruvate carboxylase deficiency***	Variable	+	+	Hyperammonemia	Severe encephalopathy
		M.A.		Elevated citrulline	Seizures
					Movement disorders
***Organic acidemias***	Prolonged fasting or after an initial symptom–free period (neonatal)	+	+	Hyperammonemia	Encephalopathy
		M.A.		Elevated branched chain amino acids and glycine	Movement disorders
				Elevated acylcarnitines	Renal disease
				UOA abnormalities	Cardiomyopathy
***Fatty acid oxidation disorders***	Fasting >8 h	+	(−)	Elevated acylcarnitines	Exercise intolerance
		M.A.	FFA/KB > 2.5	Dicarboxylic aciduria	Cardiomyopathy
				Hyperammonemia	Arrhythmias
***Ketogenesis defects***	Prolonged fasting or after an initial symptom–free period (neonatal)	+	−	Hyperammonemia	Hepatomegaly
		M.A.	FFA/KB > 2.5	Dicarboxylic aciduria	Seizures
				UOA abnormalities	Cardiomyopathy
***Ketolysis defects***	Prolonged fasting	−	+	UOA abnormalities	Intercurrent disease
			FFA/KB < 0.3		Hepatomegaly
***Disorders of Oxidative Phosphorylation***	Variable	+	+/−	UOA abnormalities	Multisystem
		M.A.			involvement
***Congenital Disorders of Glycosylation***	Variable	+/−	+/−	High insulin (mostly)	Psychomotor retardation
					Dysmorphic features
					Multisystem involvement

M.A., metabolic acidosis.

A “critical sample” (i.e. a sample obtained during HY) must be collected. Laboratory investigations should include: blood glucose, lactate, ketones (mainly beta-hydroxybutyrate), blood gases, FFA, insulin, C-peptide, cortisol, GH, Insulin, Growth Factor1 (IGF1), acylcarnitines, amino acids, as well as urinary organic acids (UOA). Some investigations may not be performed in all hospitals. Therefore, one or two spare tubes should also be collected for any additional tests to be performed afterwards. However, appropriate blood samples might be missed when an immediate treatment is required (e.g. severe HY). Still, collecting (and store frozen) the first urine sample after HY might provide helpful information in such cases (e.g. increased/undetectable ketones, lactate, tricarboxylic acids).

Additional tests can be considered: ammonia, toxicology tests, urine reducing substances test (to assess fructosuria, galactosuria). Transferrin electrophoresis/isoelectric focusing should be required if a CDG is suspected. Over the past years, a number of minimally invasive continuous glucose monitoring systems have also become available, possibly providing additional information on the extent, timing, and duration of PG fluctuations.

*Fasting test*. Evaluation of metabolic changes after fasting may be helpful to reach the diagnosis and to assess patients’ fasting tolerance to tailor the treatment. Since fasting can lead to the accumulation of toxic metabolites and sometimes fatal complications in some defects, a fasting challenge should only be performed in specialized metabolic units and only after less risky investigations have been performed without reaching a clear diagnosis (FAOD must be ruled out before a fasting test). The maximal duration of the fasting is based on the clinical suspicion and on the children’s age (usually 12–16 h at 6–12 months, 18 h at 1–2 years, 20 h at 2–7 years, 24 h in children >7 years). The fasting should be stopped at any time if glucose concentration is below 2.6 mmol/L (47 mg/dl). Since newer diagnostic strategies (biochemistry and molecular biology) are rapidly becoming available, fasting test is not performed routinely; however, it can be helpful in selected cases (96).

*Glucagon test* explores the response of glucagon injection during HY to assess the availability of glycogen for compensation of low blood glucose. Typically, HY due to GSDI does not benefit from glucagon injection (with worsening hyperlactatemia) while an exaggerated glucose response to glucagon could be observed in case of CH. Due to its possible risks (prolonged HY) it has been largely superseded by enzyme or mutation analysis.

Alternative causes of abnormality of the test results should always be ruled out (e.g. lactate elevation secondary to laborious sampling or increased pCO2 secondary to apnea during blood collection).

### Additional Investigations

Imaging tests can provide additional information. Abdominal ultrasound and Magnetic Resonance Imaging (MRI)/Computed tomography/Scintigraphy scan can define liver, spleen, pancreas, and kidneys morphology and structure (e.g. liver steatosis, liver adenomas, focal hyperplasia). Left hand and wrist X-ray can be helpful in patients with growth retardation. Specific additional investigations may be performed based on the accompanying clinical features (e.g. cardiac ultrasound, brain MRI).

## HY Comprehensive Flowchart

**Figure 2:**
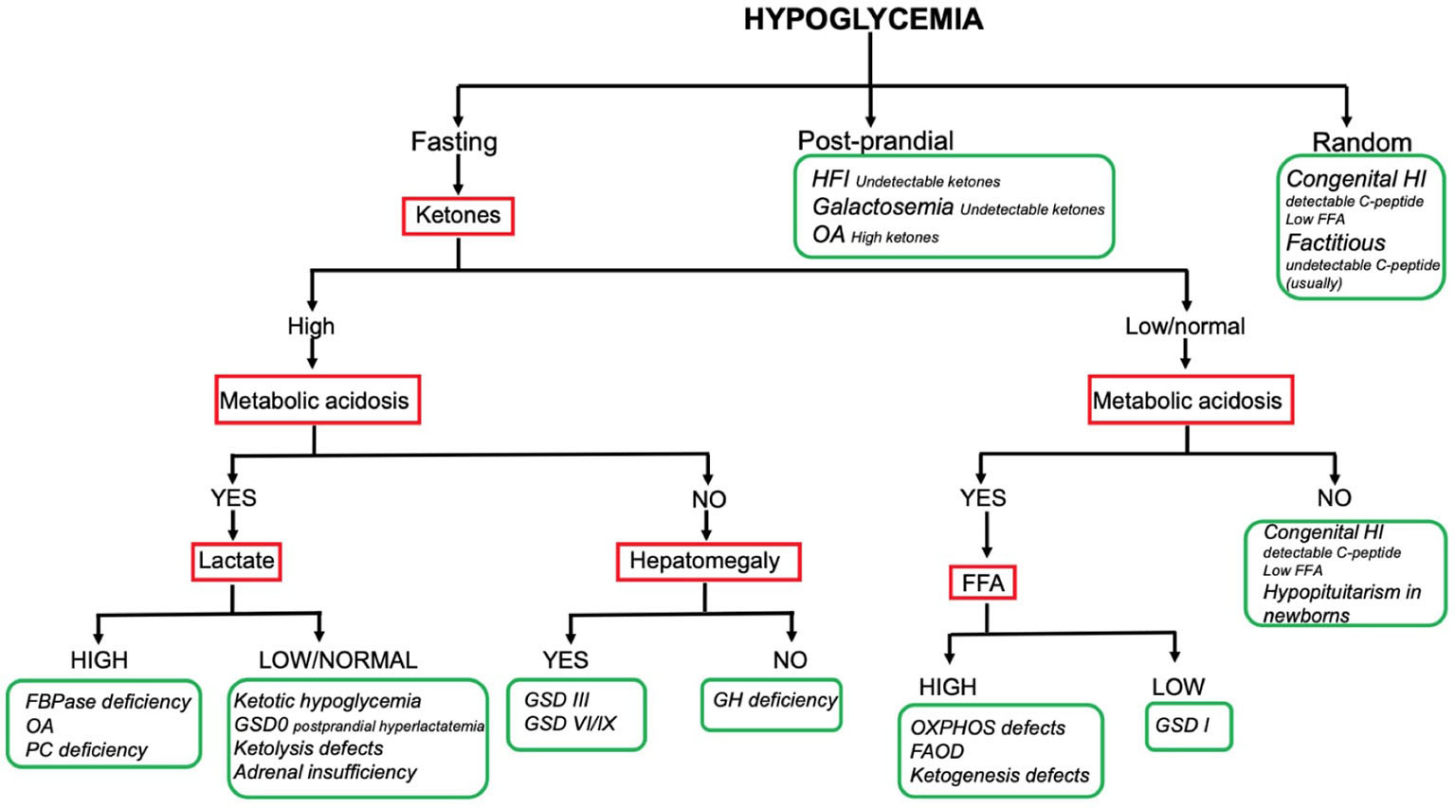
Hypoglycemia diagnostic flowchart.

The combination of the aforementioned information enables reaching a working diagnosis in most of the children presenting with HY. To date no consensus exists on a standardized diagnostic flowchart. Several algorithms with various starting points and workflow have been proposed (5, 7, 8). Although such algorithms can provide metabolic or endocrine specialists with specific pathophysiological insight, the two main groups of causes (namely endocrine and metabolic) may appear not clearly suited to the understanding and use of generalist pediatricians, which, on the other hand, are often the first level of observation of HY phenomena. Our center has a long-standing experience of cooperation between metabolic and endocrine experts in the management of childhood HY. In this respect, a comprehensive diagnostic flowchart is proposed (**Figure 2**). The major advantage of such flowchart is the ability to orient the diagnose, distinguishing both (main) metabolic and endocrine causes of childhood HY by using simple, routinely available tests such ketone bodies, emogas analysis, and lactate. As a matter of fact, data included in this flowchart can be easily implemented by physicians in a hospital setting, to obtain biochemical findings at the time of hypoglycemia, that should not be missed and that could be very useful to hypothesize the diagnosis. In order to provide adequate information, physicians should be aware that the flowchart applies to results collected on a “critical sample.” Possible limitations include the lack of rarer disorders (e.g. PEPCK deficiency, GKD, CDG) and the inability to diagnose uncommon presentations of common disorders.

Only the main diagnostic features that guide bedside diagnosis about the most common causes of pediatric HY are shown in the flowchart (e.g., hyperlactatemia is also found in OXPHOS defects and GSD I; hepatomegaly is also found in FBPase deficiency and GSD I). Firstly: the timing of HY is the starting point; the patients fasting tolerance can provide an essential clue to the diagnosis in children with fasting HY (e.g. HY after a short fast suggests hepatic GSD, HY after moderate to long fast suggests gluconeogenesis defects or FAOD/KB defects). Secondly: laboratory investigations play a pivotal role to reach a working diagnosis. In this respect, assessing the presence of (un) detectable ketones (as well as metabolic acidosis, hyperlactatemia and, if possible, FFA) on a “critical sample” is of paramount importance. Thirdly: the presence of hepatomegaly can help differentiating disorders causing fasting ketotic HY.

So far, the resultant flowchart seems to facilitate the logical process leading to the diagnostic suspicion and help to address the biochemical and clinical elements that need to be sought. The subsequent diagnostic process is up to the specialists of the two endocrine and metabolic sectors.

In case of HY in otherwise healthy children and/or with no recognizable pattern, intoxications/factitious causes should always be ruled out by toxicological tests on blood and urine (most common drugs include insulin, sulfonylurea, beta-blockers, salicylates).

Diagnosis can be confirmed through enzymatic and/or molecular testing for IMD and CH and challenge tests for endocrine disorders. Enzyme diagnostics is generally performed on blood cells or skin fibroblast (e.g. debranching enzyme or very-long chain acetyl-CoA dehydrogenase activity). However, some enzymes (e.g. G6Pase) are not expressed in these mediums and require a liver biopsy. Since liver biopsy is invasive, it has been largely superseded by DNA analysis.

## The Recent Role of NGS

DNA analysis has become increasingly sophisticated and rapid in recent years. Various techniques are used to search for mutations in IMD/CH genes; single gene analysis (Sanger sequencing) has been traditionally used to confirm a specific diagnostic suspicion, after a traditional work-up. When a group of disease is considered, the traditional diagnostic approach would involve a long process with subsequent gene-by-gene molecular analyses. The gene-by-gene technique has now been superseded and replaced by the analysis of panels with NGS techniques. The introduction of NGS represents a major advancement in the diagnostic approach, allowing in parallel sequencing of millions of small fragments of DNA. Given the difficulties in the diagnostic workup in HY and due to the overlapping of clinical manifestations in several disorders of glucose metabolism, patients showing recurrent undiagnosed HY could be further investigated with an NGS-based approach. This modern technique has the potential to identify underrecognized rare disorders in the wide group of children with ketotic hypoglycemia, clinically diagnosed in the past as affected by benign hydiopatic hypoglycemia. In addition to a targeted approach with gene panels, the NGS technology can be used through untargeted strategies based on whole‐exome sequencing, having this approach also the potential to identify new genes involved in disorders of glucose metabolism (97).

## Discussion and Conclusive Remarks

Despite being a common emergency in pediatrics (3, 4), there are still controversies on the definition and management of HY in neonates and children. Neither the standard diagnostic PG threshold nor the operative threshold are defined. Also, no consensus on the definition of at–risk neonates exists. Such uncertainties together with the broad spectrum of causes, make the approach to HY in childhood complex and time consuming. Irrespective of its cause, prompt recognition and treatment of acute HY are critical to prevent its complications (namely brain damage). Bolus administration of dextrose (either intravenously or orally) is the cornerstone of the treatment. Glucose requirements may vary depending on the patient’s age (e.g. higher in neonates) and disease (e.g. up to 10–15 mg/kg/min of glucose or more can be required in children with CH). Since additional treatments can be necessary for specific disorders (e.g. specific dietetic approach, drugs and cofactors in OA and FAOD or DZX in CH) a timely etiological diagnosis is crucial. Once acute HY has been managed, pending the results of confirmatory tests (e.g. enzyme/DNA diagnostics) specific actions should be taken in order to prevent HY relapse. As a general recommendation, fasting must be avoided and adequate carbohydrate intake must be maintained during any metabolic stress. Tailored dietary treatment plan with frequent feedings and UCCS and/or tube feeding are the most common interventions, such a plan aims at ensuring glucose concentrations as stable as possible and is generally sufficient in patients with ketotic HY, hepatic GSD, and disorders of KB metabolism. Additional dietary interventions may be required for specific IMD (e.g. life–long fructose–, sucrose– sorbitol–restricted diet regimen in HFI, or low–protein diet in OA). Irrespective of their final diagnosis, in acute situations (e.g. intercurrent illness, prolonged fasting) patients can become catabolic, due to (the combination of) high fever, a reduced intake, and/or increased losses. Therefore, it is important to know what to do in emergency situations. An emergency protocol is designed at this purpose (98). Patients (and caregivers) should be encouraged to always carry an emergency protocol with them and follow its instructions. As HY can be secondary to a variety of different disorders, a systematic multidisciplinary approach is ideal in caring for neonates and children with HY. Interestingly, there is no consensus on standardized diagnostic algorithms for childhood HY. Therefore, a comprehensive practical diagnostic flowchart (including the main endocrine and metabolic causes) is proposed to guide the diagnostic suspicion, highlighting a minimal set of clue clinical and biochemical findings at the time of HY, that can be easily investigated in any hospital, at any time of the day. In fact, it is of paramount importance that samples are collected during HY (i.e. “critical sample”), otherwise the diagnosis can be missed (biochemical investigations might result normal when euglycemia has been reached). As shown in the proposed diagnostic flowchart, the minimal set of biochemical findings in children presenting with of HY includes ketones, lactate (both in blood and urine), and blood gases (i.e. metabolic acidosis). Such findings can help reaching a provisional diagnosis, which can be confirmed with additional (biochemical and/or genetic) tests. In this respect, collecting (and store adequately) additional samples at the time of HY is crucial. Laboratory data must also be appropriately integrated with anamnestic, dietary, clinical, and imaging information. The proposed flowchart aims at guiding the diagnostic management of a such common manifestation in pediatric age that can be due to a wide spectrum of causes. A double level usefulness is expected for the proposed flowchart: the first one is addressed to general pediatrician by providing the clinical–anamnestic and laboratory findings to be sought in order to refer the patient to the most appropriate Tertiary Center (Endocrinological/Metabolic/Genetic disease), the second level is for specialists in pediatric endocrine–metabolic diseases in order to remind them the wider etiological spectrum of pediatric HY by giving the essential elements of the differential diagnosis involving different areas (genetic, endocrine, and metabolic). In other word our flowchart aims to be a quick scheme to help pediatricians of every setting in managing HY, attempting to be comprehensive of the main disorders and differential diagnosis. Of course, mostly compared to the current available flowcharts focused on peculiar fields (metabolic or endocrinological), some rare conditions or rare presentations cannot be included. Sometimes, clinicians are not able to reach a final diagnosis, despite multiple efforts, due to the lack of specific biochemical pattern or atypical presentation of some disorders. In such cases, innovative diagnostic techniques can be considered. Even if much progress has been made over past years, many things remain to be discovered and clarified for diseases causing HY in childhood. Advances in diagnostic techniques (e.g. NGS) will identify specific defects or even new entities in a subgroup of patients who have been diagnosed with ketotic HY, likely resulting in a change in the disease epidemiology or in the discovery of new conditions (97).

In conclusion, future studies are also needed to optimally define normal glucose thresholds in neonates and children (10). In addition, irrespective of the specific diagnosis prompt recognition and treatment of acute HY are critical to prevent its complications, the diagnostic work–up should start at the emergency hospital, collecting critical sample at the time of HY and providing specialists the clued results from simple tests that are available at any hospital at any time and that are very useful to address the clinical suspicion.

Based on the recognized risks of some tests the traditional diagnostic process, including fasting or dynamic tests, is presently controversial and probably superseded by modern molecular diagnostic techniques. NGS approach has also the potential to diagnose disorders with mild biochemical abnormalities or atypical presentations or even to identify new diseases, changing the epidemiology of many disorders. In this respect, the development of extended collaboration networks for rare diseases is worthy (43).

## Author Contributions

AC, AR and EM wrote the manuscript. EM, AF, SF and GP reviewed the manuscript. FMR, CM and FDC edited the manuscript and collected data. EM and SF are the guarantors of this work and, as such, had full access to all the data in the study and take responsibility for the integrity of the data and the accuracy of the data analysis. All authors were responsible for drafting the article and revising it critically for important intellectual content. All authors contributed to the article and approved the submitted version.

## Conflict of Interest

The authors declare that the research was conducted in the absence of any commercial or financial relationships that could be construed as a potential conflict of interest.

## Publisher’s Note

All claims expressed in this article are solely those of the authors and do not necessarily represent those of their affiliated organizations, or those of the publisher, the editors and the reviewers. Any product that may be evaluated in this article, or claim that may be made by its manufacturer, is not guaranteed or endorsed by the publisher.

## Abbreviations

AAP, American Academy of Pediatrics; ACTH, Adreno–CorticoTropic Hormone; AI, Adrenal Insufficiency; BAPM, British Association for Perinatal Medicine; CAH, Congenital Adrenal Hyperplasia; CAI, Central Adrenal Insufficiency; CDG, Congenital Disorders of Glycosylation; CH, Congenital Hyperinsulinism; CoA, Coenzyme A; CPT, Carnitine Palmitoyl–Transferase; CRH, Corticotropin–Releasing Hormone; DZX, Diazoxide; FAOD, Fatty Acid Oxidation Disorders; FBPase, Fructose 1,6 bisphosphatase; FFA, Free Fatty Acids; G6Pase, Glucose–6–Phosphatase; GALT, Galactose–1–phosphate uridyltransferase; GDH, Glutamate Dehydrogenase Enzyme; GH, Growth Hormone; GHD, Growth Hormone Deficiency; GHRH, Growth Hormone–Releasing Hormone; GKD, Glycerol Kinase Deficiency; GLP–1, Glucagon–Like Peptide–1; GSD, Glycogen Storage Disease; HFI, Hereditary Fructose Intolerance; HY, Hypoglycemia; IGF1, Insulin Growth Factor1; IMD, Inborn Metabolic Disorder; IVA, Isovaleric Acidemia; KB, Ketone Bodies; LGA, Large for Gestational Age; MMA, Methylmalonic Acidemia; MRI, Magnetic Resonance Imaging; NBS, Newborn Screening; NGS, Next Generation Sequencing; OA, Organic Acidemias; OXPHOS, Oxidative Phosphorylation; PA, Propionic Acidemia; PAI, Primary Adrenal Insufficiency; PC, Pyruvate Carboxylase; PEPCK, Phosphoenolpyruvate Carboxykinase; PES, Pediatric Endocrine Society; PET, Positron Emission Tomography; PG, Plasma Glucose; SGA, Small for Gestational Age; UCCS, Uncooked cornstarch; UDP, Uridine Di–Phosphate; UOA, Urine Organic Acids.
