# Supplementary material for: An Overview of Hypoglycemia in Children Including a Comprehensive Practical Diagnostic Flowchart for Clinical Use
Source: Front Endocrinol (Lausanne). 2021 Aug 2;12:684011. doi: 10.3389/fendo.2021.684011 (PMC8366517; doi:10.3389/fendo.2021.684011)
Supplement: Supplementary file 1 [file Table_1.pdf]

frontiers

Supplemental table 1. Major IMD groups associated with hypoglycaemia. Most common Glycogen storage diseases, Fatty acid oxidation disorders and congenital disorders of glycosylation are included.

**Disorder**	**Gene(s)**	**Protein**	**Inheritance**
GSD0a	*GYS2*	Liver glycogen synthase	Autosomal recessive
GSD0b	*GYS1*	Muscle glycogen synthase	Autosomal recessive
GSDIa	*G6PC*	Glucose 6-phosphatase α	Autosomal recessive
GSDIb	*SLC37A4*	Glucose 6-phosphate transporter	Autosomal recessive
GSDII (Pompe disease)	*GAA*	lysosomal α-glucosidase	Autosomal recessive
GSDIII	*AGL*	Glycogen debrancher enzyme	Autosomal recessive
GSDIV	*GBE1*	Glycogen brancher enzyme	Autosomal recessive
GSDV	*PYGM*	Muscle glycogen phosphorylase	Autosomal recessive
GSDVI	*PYGL*	Liver glycogen phosphorylase	Autosomal recessive
GSDVII	*PFKM*	Muscle phosphofructokinase	Autosomal recessive
GSDIXa	*PHKA2*	Glycogen phosphorylase kinase α2	X-linked recessive
GSDIXb	*PHKB*	Glycogen phosphorylase kinase β	Autosomal recessive
GSDIXc	*PHKG2*	Glycogen phosphorylase kinase γ	Autosomal recessive
GSDIXd	*PHKA1*	Glycogen phosphorylase kinase α1	X-linked recessive
GSDX	*PGAM2*	Muscle phosphglycerate mutase	Autosomal recessive
GSDXI (Fanconi-Bickel)	*SLC2A2*	GLUT2	Autosomal recessive
GSDXII	*ALDOA*	Aldolase A	Autosomal recessive
GSDXIII	*ENO3*	β-enolase	Autosomal recessive
GSDXIV	*PGM1*	Phospoglucomutase	Autosomal recessive
GSDXV	*GYG1*	Muscle glycogenin deficiency	Autosomal recessive
Hereditary fructose intolerance	*ALDOB*	Aldolase B	Autosomal recessive
Classical galactosemia	*GALT*	Galactose-1p uridyltransferase	Autosomal recessive
Fructose 1,6-Bisphosphatase deficiency	*FBP1*	Fructose 1,6-Bisphosphatase	Autosomal recessive
Pyruvate carboxylase deficiency	*PC*	Pyruvate carboxylase	Autosomal recessive
Methylmalonic acidemia	*MMUT, MMA A, MMAB, MMA DHC, MCEE*	Methylmalonil-CoA mutase, Methylmalonil-CoA epimerase	Autosomal recessive

Propionic acidemia	*PCCA, PCCB*	Propionyl-CoA carboxylase	Autosomal recessive
Isovaleric acidemia	*IVD*	Isovaleryl-Coa dehydrogenase	Autosomal recessive
Carnitine transporter deficiency	*SLC22A5*	Carnitine transporter	Autosomal recessive
Carnitine palmitoyltransferase I (CPT I) deficiency	*CPT1*	Carnitine palmitoyltransferase I	Autosomal recessive
Carnitine acylcarnitine translocase (CACT) deficiency	*SLC25A20*	Carnitine acylcarnitine translocase	Autosomal recessive
Carnitine palmitoyltransferase II (CPT II) deficiency	*CPT2*	Carnitine palmitoyltransferase II	Autosomal recessive
Very-long-chain acyl-CoA dehydrogenase (VLCAD) deficiency	*ACADVL*	Very-long-chain acyl-CoA dehydrogenase	Autosomal recessive
Long-chain 3-hydroxyacyl-CoA dehydrogenase (LCHAD)<br>and mitochondrial trifunctional protein (MTP) deficiency	*HADHA, HADHB*	Long-chain 3-hydroxyacyl-CoA dehydrogenase,Long-chain enoyl-CoA hydratase, Long-chain ketoacylCoA thiolase	Autosomal recessive
Medium-chain acyl-CoA dehydrogenase (MCAD) deficiency	*ACADM*	Medium-chain acyl-CoA dehydrogenase	Autosomal recessive
Short-chain acyl-CoA dehydrogenase (SCAD) deficiency	*ACADS*	Short-chain acyl-CoA dehydrogenase	Autosomal recessive
3-Hydroxyacyl-CoA dehydrogenase (SCHAD) deficiency	*HADH*	3-hydroxyacyl-CoA dehydrogenase	Autosomal recessive
Multiple acyl-CoA dehydrogenase (MAD) deficiency	*ETFA, ETFB, ETFDH*	Electron transfer flavoproteins	Autosomal recessive
Riboflavin metabolism defects	*SLC52A1, SLC52A2, SLC52A3, FLAD1, SLC25A32*	RFVT1, RFVT2, RFVT3, FAD synthase,<br>FAD transporter	Autosomal recessive
3-Hydroxy-3-Methylglutaryl-CoA (HMG-CoA) Synthase Deficiency	*HMGCS2*	3-Hydroxy-3-Methylglutaryl-CoA synthase	Autosomal recessive
HMG-CoA Lyase Deficiency	*HMGC2*	3-Hydroxy-3-Methylglutaryl-CoA lyase	Autosomal recessive
Succinyl-CoA:3-oxoacid CoA transferase (SCOT) deficiency	*OXCT1*	Succinyl-CoA:3-oxoacid CoA transferase	Autosomal recessive
Mitochondrial acetoacetyl-CoA thiolase (T2) deficiency	*ACAT1*	Acetoacetyl-CoA thiolase	Autosomal recessive
Monocarboxylate transporter 1 (MCT1) deficiency	*SLC16A1*	Monocarboxylate transporter 1	Autosomal recessive
OXPHOS disorders	*Multiple genes*	Respiratory complexes subunits	Variable
Phosphomannose isomerase (MPI-CDG) deficiency	*PMI*	Phosphomannose isomerase	Autosomal recessive
Phosphomannomutase 2 (PMM2-CDG) deficiency	*PMM2*	Phosphomannomutase 2	Autosomal recessive
